# Politicizing the Precautionary Principle: Why Disregarding Facts Should Not Pass for Farsightedness

**DOI:** 10.3389/fpls.2019.01053

**Published:** 2019-08-26

**Authors:** Philipp Aerni

**Affiliations:** Center for Corporate Responsibility and Sustainability (CCRS), University of Zurich, Zurich, Switzerland

**Keywords:** gene-editing, regulatory approval process, precautionary principle, GMO (Genetically modified organism), sustainable development goal (SDG)

## Introduction

In his seminal book “The Death of Expertise” Thomas Nichols (2017) explores how “ignorance became a virtue” (Kakutani, 2017) in public debates on controversial issues in the United States, revealing a growing hostility toward scientific expertise. A similar trend can be observed in Europe, especially when it comes to the regulation of agricultural biotechnology.

In response to widespread public concerns about the potential risks of genetically modified organisms (GMOs) in food and agriculture, the EU legislative bodies passed Directive 2001/18/EC on the deliberate release of GMO into the environment in the year 2001.^1^ As for the history of safe use, this so-called GMO Directive implies that there is a fundamental difference between crops improved by means of genetic engineering and crops improved by any other established types of breeding technology, including classic mutagenesis, which is widely considered to be a more uncertain manipulation of the plant DNA than genetic engineering (SAM and High-level Group of Scientific Advisors, 2017). The GMO Directive is meant to follow the Precautionary Principle (PP), which has been defined in detail by the European Commission (EC) in its “Communication on the Precautionary Principle,” published in the year 2000 (EC (European Commission), 2000). It states that the PP should adhere to the general principles of risk management, which include (a) the principle of proportionality between the measures taken and the chosen level of protection; (b) the principle of nondiscrimination in the application of the measures; (c) consistency of the measures with similar measures already taken in similar situations; (d) the examination of the benefits and costs of action or lack of action; and (e) review of the measures in the light of scientific developments. This interpretation of the PP is scientifically sound and has a long track record in national and international environmental policy. Yet, by treating genetic engineering as an environmental risk in a broad sense, the GMO Directive has more in common with toxic waste regulation than with the registration of a new plant variety (Sprankling and Salcido, 2018). As such, the new regulation did not help address the EU’s de-facto moratorium on biotech products in place since 1998 and thus induced major exporters of GM crops to submit a first request for consultation with the World Trade Organization (WTO) on May 13, 2003, on the consistency of the GMO regulation in Europe with WTO rules.

In 2006, the dispute settlement panel of the WTO took a decision on the case “European Communities Measuring and Affecting the Approval and Marketing of Biotech Products” (DS291, 292, 293). The panel faulted the European Union for causing undue delay in the approval of biotech products and pointed at the fact that the additional safeguard measures applied by EU member states were not based on proper risk assessment as required by the WTO Agreement on the Application of Sanitary and Phytosanitary Measures (SPS Agreement). The SPS Agreement endorses the use of the PP as long as it is combined with an effort to gain more science-based information on the potential risks and eventually adjust regulation correspondingly.^2^ The EU and its member states made such an effort by spending hundreds of millions of Euros on risk research on GMOs (EC (European Commission), 2010); yet, they failed to take any action based on the insights gained from this research. The main finding, which was also reaffirmed by all national academies worldwide, was that there are indeed risks, but that these risks are also well known in conventional agriculture. As a result, the EU should have taken appropriate action to better align its regulatory approach to GMOs with the risk management principles that underpin the PP. But for that purpose, risk management would have to shift from a process-based to a product-based approach of risk assessment. This has not happened because the PP ceased to be a tool of responsible risk management, but instead became a convenient excuse to postpone approval decisions by pointing out that off-target effects in the breeding process and indirect adverse effects resulting from the commercial use of GMO cannot be excluded entirely; however, the likelihood of such effects to occur often turns out to be lower in the case of GMO than with unregulated classical mutagenesis or conventional breeding due to the higher degree of precision and efficiency of advanced biotechnology, the more accurate identification of off-target effects, and the more strict monitoring requirements (Lazebnik et al., 2017; SAM and High-level Group of Scientific Advisors, 2017:58).

Despite the highly preventive EU regulatory framework, a few GM crops, being considered “safe,” eventually won temporary approval for cultivation in the EU; but many EU member states continued to prohibit them in their territories claiming “safety concerns.” In response to this disregard of EU regulation, the EC proposed in 2015 to amend the legislation so that Member States are free to restrict or prohibit the cultivation of EU-authorized GM crops on their territory on the basis of grounds that divert from those assessed by the harmonized set of Union rules as outlined in Directive 2001/18/EC and Regulation (EC) No. 1829/2003 on GM food and feed. By including broadly defined “social concerns,” the resulting Directive (EU) 2015/412^3^ has the effect that the application of the PP ceases to be limited to potential danger for which there is credible scientific evidence (Hansson, 2016).

Lawmakers and judges in the EU nevertheless continue to invoke the PP as justification for banning GMOs (Alemanno, 2007; Lamping, 2012; Heubuch, 2016). These preventive measures may not be unpopular since numerous advocacy groups concerned with the environment, sustainable agriculture, and consumer interests will praise them as being farsighted.

The disregard of the principles that underpin the PP is not just a phenomenon among politicians with a clear antibiotech agenda, but also prevalent in the field of ethics. In the Report on the Precautionary Principle, published by the Swiss Federal Ethics Committee on Non-Human Biotechnology (EKAH (Swiss Federal Ethics Committee on Non-Human Biotechnology), 2018) in spring 2018, the committee members point at the ethical foundations behind the principle and describe it as a tool to protect society from potentially harmful consequences of scientific and technological advances. The cover page of the report features Pandora’s Box as a symbol for all the evils that may result from the advances in modern plant breeding. Suggestively, it visually links the risks of genetic engineering with the risks of nuclear plants, toxic waste, and oil spills and contrasts it with pictures of healthy Swiss agricultural landscapes and happy farmers. Unsurprisingly, the committee reaches the conclusion that the new breeding techniques (NBTs) that involve gene editing should be regulated like genetic engineering in food and agriculture in order to protect society and the environment. The committee does not refer to the safe track record of existing GMOs in the market, nor does it cite the recent detailed expert assessment by the Science Advisory Group of the EC (SAM and High-level Group of Scientific Advisors, 2017) of the different gene-editing techniques. Moreover, it does not address the ethical issues related to the instrumental use of the PP for political ends, especially by lobbying groups that benefit from the status quo (Aerni, 2018). In this sense, the EKAH report once again treats the PP as a tool to make disregard look like far-sightedness and, as such, anticipates the decision of the European Court of Justice (ECJ) on July 25, 2018.

The ECJ issued an explanation toward the French High Court (Conseil d’Etat) as to what extent NBT falls under the category of GMO as defined by Directive 2001/18/EC.^4^ It stated that organisms obtained by NBT are to be considered GMOs. This would also apply to point mutations generated by NBT as they would not fall under the express “GMO”^5^ exemption of mutagenesis in Annex 1B of the Directive comprising conventional techniques of mutagenesis that would have a long safety record. Unlike the report of the Swiss ethics committee, the ruling of the ECJ may have serious consequences for the future of science in Europe. By interpreting Directive 2001/18/EC in a way that would subject NPT to GMO regulation, the ruling may render the cultivation of crops that have been bred with even the least invasive forms of gene-editing in a limbo of legal uncertainty in Europe. As for the imports of such crops into the European Union from countries that have already decided to not subject NBT to the same burdensome regulations of GMOs, such as the United States, Argentina, or Chile (Eriksson et al., 2019), there will be great technical and political difficulties to ensure the same costly separation and corresponding labeling of bulk agricultural commodities. The EC’s Joint Research Centre confirms that it will be impossible to understand if a point mutation derives from a spontaneous event or a human intervention (Emons et al., 2018). As a consequence, the European rapid alert system^6^ might collapse.

The European retailers, which campaigned in advance of the ECJ decision to subject gene-editing techniques to the same regulation as GMO,^7^ may have been aware of this. But, as consumer choice is driven by affect rather than deliberate reasoning (Aerni, 2011; Stasi et al., 2018), retailers tend to make use of GMO-free labeling strategies that cultivate consumer fears rather than point at the long safety record of GMOs for human consumption (Ray and Wilkie, 1970; Laros and Steenkamp, 2004; Schurman, 2004; Aerni et al., 2011; Russo, 2015).

But, maybe, it is neither the EKAH, ECJ, the anti-GMO activists, nor the retailers that are to blame for the widespread disregard of the facts about modern biotechnology. Instead, it is the old Directive 2001/18/EC and its definition of a GMO. In Article 2, GMO is defined as “an organism, with the exception of human being, in which the genetic material has been altered in a way that does not occur naturally by mating and/or natural recombination.” If that definition would be followed with the ECJ to its extremes, then consumer choice in supermarkets would probably shrink to a tiny number of wild fruits, vegetables, and cereals. In return, it has also been shown that many of the common types of alterations introduced by NBT could also potentially occur naturally, if “occurring naturally” includes conventional breeding too, as the Directive 2001/18/EC seems to imply (Custers et al., 2018).

The inconsistent use of the term GMO and, with it, NBT in Europe (Ammann, 2014; Tagliabue, 2016) may eventually lead to prohibitive regulation in many other countries and thus become a serious obstacle to the Agenda 2030, the implementation plan to achieve the Sustainable Development Goals (SDGs). The SDGs were approved by the United Nations General Assembly in fall 2015 with the purpose of creating a more inclusive and sustainable global community by 2030. At the core of the SDGs is global agriculture. It will have to increase the quantity and the quality of food production in order to ensure greater access to healthy diets. Simultaneously, agriculture must become more sustainable by reducing the use of fertilizer and means of plant protection. The combination of objectives can only be achieved by means of sustainable intensification, which includes the genetic improvement of plants so that they become more tolerant to biotic and abiotic stress factors, make better use of photosynthesis and soil nutrients, and enhance the nutritional value of basic food crops. Conventional plant breeding may still be able to address some of these challenges, but it is time-consuming and cannot be tailored well to local preferences, which results in low adoption rates (Aerni, 2006). New breeding techniques have the potential to address these drawbacks. In this context, the PP, based on the Commission’s own definition (EC (European Commission), 2000), would be obliged to also assess the risk of nonaction (Aerni et al., 2016). This is also the view of the Group of Chief Scientific Advisors of the EC, which challenges the ruling of the ECJ in a statement published in November 2018 in which it regards the old GMO Directive as no longer fit for purpose (GCSA (Group of Chief Scientific Advisors), 2018).

However, as long as the current process-based regulation continues to be defended by leading European stakeholders from an ethical, legal, and retail business perspective and in disregard of scientific expertise, Europe will be unable to meet its own ambitions to contribute to the numerous SDGs through the creation of a sustainable bioeconomy (Aerni, 2018; EC, 2018).

## Author Contributions

PA is the single author of this contribution.

## Funding

No funding has been received for doing the research on this article. CCRS is an associated institute of the University of Zurich with base-funding from the University of Zurich and the Zurich Cantonal Bank.

## Conflict of Interest Statement

The author declares that the research was conducted in the absence of any commercial or financial relationships that could be construed as a potential conflict of interest.

## Footnotes

1 Directive 2001/18/EC was an amendment of Council Directive 90/220/EEC on the deliberate release into the environment of GMOs, passed in 1990 (see http://eur-lex.europa.eu/LexUriServ/LexUriServ.do?uri=CELEX:31990L0220:EN:NOT).

2
https://www.wto.org/english/tratop_e/dispu_e/cases_e/ds291_e.htm (visited on February 12, 2019).

3
https://eur-lex.europa.eu/legal-content/EN/TXT/?uri=celex%3A32015L0412 (visited on February 12, 2019).

4 InfoCuria—Case-law of the Court of Justice. Case Number C-528/16.

5 The ECJ follows Directive 2001/18/EC by regarding organisms obtained by means of techniques/methods of mutagenesis as genetically modified organisms, yet they are explicitly excluded from the scope of the Directive and therefore not subject to GMO regulation. But if any Member State will ask for the repeal of this rather contrived exclusion, even classical mutagenized plants may have to go through the same regulation like GMOs. Such a decision may be impossible to implement as all the 3301 mutagenized plants (https://mvd.iaea.org/), and any other deriving from a cross with them, would have to go through the burdensome GMO approval process.

6
https://ec.europa.eu/food/safety/rasff_en (visited on June 27, 2019).

7
https://www.ohnegentechnik.org/fileadmin/ohne-gentechnik/presse/p_180710_Offener_Brief_EU_Kommission_180710.pdf (visited on July 29, 2019).
